# Human single-unit recordings reveal a link between place-cells and episodic memory

**DOI:** 10.3389/fnsys.2014.00158

**Published:** 2014-08-29

**Authors:** Johannes Niediek, Jonathan Bain

**Affiliations:** ^1^The Edmond and Lily Safra Center for Brain Sciences, Hebrew UniversityJerusalem, Israel; ^2^Department of Epileptology, University of BonnBonn, Germany

**Keywords:** hippocampus, place-cells, navigation, episodic memory, human single-unit

Human episodic memory refers to our ability to remember personal experiences including mentally reliving such an experience; remembering what happened; and knowing where and when it happened. Evidence from amnesic patients shows that the hippocampus is a necessary brain structure for episodic memory (Scoville and Milner, 1957). Its role in episodic memory has been studied extensively in fMRI experiments (for a review, see e.g., Eichenbaum et al., 2007) but to date no mechanism has been identified to explain how neuronal ensembles enable encoding and retrieval of memory episodes. An interesting hint comes from rodent studies: in the hippocampi of rats and mice, researchers found “place-cells,” which are spatially selective cells. These cells show an increase in firing rate when the animal moves through a specific part of an arena (O'Keefe and Dostrovsky, 1971).

In humans, place-cells have been found in the hippocampus and other sub-regions of the medial temporal lobe (MTL) using virtual navigation (Ekstrom et al., 2003). A recent comparison between real-world and virtual navigation in rats showed robust hippocampal place-cells in both types of navigation, but some differences in coding properties and place-field sizes were also observed (Ravassard et al., 2013).

It has been speculated that the spatial component of episodic memories may be encoded by place-cell activity. To study this idea, Miller et al. (2013) attempted to link place-cell activity with episodic memory by means of its spatial context. The researchers suggest that the same brain mechanisms that represent space and contribute to navigation are also active in the encoding and retrieval of episodic memory.

The researchers recorded single- and multi-unit activity from the hippocampus and surrounding MTL areas of seven epilepsy patients using depth electrodes, which were implanted for clinical reasons. Navigating a virtual city, the patients had to visit 13 different locations. At each of the first 12 locations they encountered an object, and at the final location they had to recall the objects. Close to 26 percent of the recorded cells were classified as place-cells (95 of 371). The researchers found place-cells in the hippocampus, amygdala, entorhinal cortex and anterior MTL. Seventy-two percent (68 of 95) of the recorded place-cells were direction-dependent.

The researchers then investigated, whether the cells' activity during recall resembled that during navigation. They divided the city into 100 × 100 squares and calculated a response vector for each square, representing the neuronal ensemble firing as the patient navigated throughout the entire city. Then, for each object, the researchers compared the ensemble activity during recall to the respective square's response vector and computed a similarity score. They found that the similarity scores were highest in regions defined as “near” the location where the object was encountered, and lower at “far” locations. They also found a moderate increase in place-cell firing rates during recall for the “near” condition, compared to the ‘far’ condition. These findings demonstrate that place-cells show similar patterns of activity for traveling through locations in space as for memory recall related to those locations.

Our knowledge of place-cells in humans is still limited and the authors of this study collected a vast amount of highly relevant data. We believe the experimental paradigm is well suited to testing the role of place-cells in episodic memory. However, we would like to suggest that the publication nonetheless demonstrates several potential shortcomings.

The researchers presented only two “place-cell heat-maps.” In comparison, Wilson and McNaughton (1994) showed more than 100 different heat-maps of place-fields in rats. The description of the spatial representation in the neuronal recordings is incomplete with such a low number of example place-fields and without their distribution across the arena. Additionally, plots of subjects' paths through the environment along with spike raster plots for repeatedly visited locations would also need to be included to demonstrate the reliability of place-cells. Furthermore, a description of the place-fields' typical sizes and shapes is missing, which seems relevant as the two published place-fields show concave shapes. One potential source for such irregular shapes can be the methodology used to identify place-fields. The authors classified a cell as being place-responsive if its mean firing rate in a contiguous, sufficiently large region differed significantly from its firing rates elsewhere. To our understanding, this condition does not rule out disconnected or scattered place fields.

The authors reported on average 12, and up to 26, units in the hippocampus, using 8–32 micro-electrodes. Such high numbers suggest that the raw data quality must have been extraordinarily good (according to author M.J.K. Misra et al., 2014 less than 3 units per 8 micro-electrodes can be recorded). The field of human single-cell recordings is beginning to embrace standards already in use in animal research, such as presenting raw data traces and overlay-plots of spikes after sorting (e.g., Rutishauser et al., 2011), or plots of selected cluster features to confirm cluster separation (e.g., Lee and Wilson, 2002). It is also important to report the effect of spike-sorting on the main findings. Given the high unit yield, it is conceivable that spike-sorting had substantial influence on the distribution, shapes, and sizes of the reported place-fields. We believe that such additional information is necessary for correct interpretation of these highly relevant recordings.

The authors of the study also analyzed the reinstatement of spatially informative neural activity by parametric hypothesis testing. An additional, possibly clearer analysis would have been the prediction of the objects' locations based on the recall responses (or similarity scores), similar to an analysis by Davidson et al. (2009). The predicted locations could be imposed onto a map of the arena, thus giving a comprehensive overview of how well spatial context can be extracted from neural activity during recall.

We think that the study by Miller et al. contributes important data to episodic memory research. More extensive analysis and presentation of this rare data would allow for a more thorough understanding of the MTL's role in human navigation and memory. We agree with the authors, that such arduous and costly research can reveal insights that cannot be gleaned from non-human studies alone.

## Conflict of interest statement

The authors declare that the research was conducted in the absence of any commercial or financial relationships that could be construed as a potential conflict of interest.

## References

[B1] Davidson T. J. Kloosterman F. Wilson M. A. (2009). Hippocampal replay of extended experience. Neuron 63, 497–507 10.1016/j.neuron.2009.07.027 19709631 PMC4364032

[B2] Eichenbaum H. Yonelinas A. P. Ranganath C. (2007). The medial temporal lobe and recognition memory. Ann. Rev. Neurosci. 30, 123–152 10.1146/annurev.neuro.30.051606.094328 17417939 PMC2064941

[B3] Ekstrom A. D. Kahana M. J. Caplan J. B. Fields T. A. Isham E. A. Newman E. L. (2003). Cellular networks underlying human spatial navigation. Nature 425, 184–188 10.1038/nature01964 12968182

[B4] Lee A. K. Wilson M. A. (2002). Memory of sequential experience in the hippocampus during slow wave sleep. Neuron 36, 1183–1194 10.1016/S0896-6273(02)01096-6 12495631

[B5] Miller J. F. Neufang M. Solway A. Brandt A. Trippel M. Mader I. (2013). Neural activity in human Hippocampal formation reveals the spatial context of retrieved memories. Science 342, 1111–1114 10.1126/science.1244056 24288336 PMC4669102

[B6] Misra A. Burke J. F. Ramayya A. G. Jacobs J. Sperling M. R. Moxon K. A. (2014). Methods for implantation of micro-wire bundles and optimization of single/multi-unit recordings from human mesial temporal lobe. J. Neural Eng. 11, 026013 10.1088/1741-2560/11/2/026013 24608589 PMC4019382

[B7] O'Keefe J. Dostrovsky J. (1971). The Hippocampus as a spatial map. Preliminary evidence from unit activity in the freely-moving rat. Brain Res. 34, 171–175 5124915 10.1016/0006-8993(71)90358-1

[B8] Ravassard P. Kees A. Willers B. Ho D. Aharoni D. Cushman J. (2013). Multi-sensory control of Hippocampal spatiotemporal selectivity. Science 340, 1342–1346 10.1126/science.1232655 23641063 PMC4049564

[B9] Rutishauser U. Tudusciuc O. Neumann D. Mamelak A. N. Heller A. C. Adolphs R. (2011). Single-Unit responses selective for whole faces in the human Amygdala. Cur. Biol. 21, 1654–60 10.1016/j.cub.2011.08.035 21962712 PMC4574690

[B10] Scoville W. B. Milner B. (1957). Loss of recent memory after bilateral Hippocampal lesions. J. Neurol. Neurosurg. Psychiatry 20, 11–21 13406589 10.1136/jnnp.20.1.11 PMC497229

[B11] Wilson M. A. McNaughton B. L. (1994). Reactivation of Hippocampal ensemble memories during sleep. Science 265, 676–679 8036517 10.1126/science.8036517

